# Optimization of Fermentation Conditions for Endophytic Fungi from *Schisandra chinensis* and Investigation of Their Antibacterial Mechanisms Against Methicillin-Resistant *Staphylococcus aureus*

**DOI:** 10.3390/microorganisms13050982

**Published:** 2025-04-25

**Authors:** Mengyu Li, Yuewei Shi, Wenwei Ma, Shouyuan Cai, Xinyuan Yang, Lukai Xu, Xiyan Hou, Lulu Wang, Liming Jin, Chunshan Quan

**Affiliations:** Key Laboratory of Biotechnology and Bioresources Utilization of Ministry of Education, College of Life Sciences, Dalian Minzu University, Dalian 116600, China; 15825315619@163.com (M.L.); 19841140811@163.com (Y.S.); 15842436127@139.com (W.M.); 18265502153@163.com (S.C.); syyy-@outlook.com (X.Y.); xvlukai@163.com (L.X.); xyhous@dlnu.edu.cn (X.H.); wanglulu0813@126.com (L.W.)

**Keywords:** MRSA, *Rhizoctonia bicornis*, *Schisandra chinensis*, antibacterial mechanism

## Abstract

Methicillin-resistant *Staphylococcus aureus* (MRSA) is a highly pathogenic bacterium for which safe and effective prevention as well as treatment methods are currently lacking. In this study, *Rhizoctonia bicornis* S10 was isolated from *Schisandra chinensis* and used to inhibit MRSA. Through single-factor experiments and response surface methodology, the optimal culture medium and fermentation conditions for *Rhizoctonia bicornis* S10 were determined. The results demonstrated that optimal growth conditions for *Rhizoctonia bicornis* S10 were achieved using corn meal as the culture medium. Furthermore, the extract of *Rhizoctonia bicornis* S10 showed maximal inhibitory activity against MRSA under the following optimized conditions: moisture content of 150%, time of 2.5 d, temperature of 30 °C, and pH 5.0. Antagonistic experiments demonstrated that the crude extract of *Rhizoctonia bicornis* S10 exhibits significant inhibitory activity against MRSA. During the growth of MRSA, the crude extract effectively suppressed its proliferation. Measurements of alkaline proteinase and membrane potential confirmed the disruption of the MRSA cell membrane, which was further visually validated by scanning electron microscopy. Additionally, the crude extract was shown to inhibit the formation of MRSA biofilms. These findings suggest that *Rhizoctonia bicornis* S10 should hold promise as a novel approach for the prevention and treatment of MRSA infections, providing new strategies and insights for combating this pathogen.

## 1. Introduction

Since the discovery of penicillin by British scientist Alexander Fleming in 1929, research into antibiotics has significantly progressed. The advent of these antimicrobial agents has enabled clinicians to manage life-threatening infections effectively. However, the overuse and misuse of antibiotics have led to the emergence of drug-resistant bacteria.

*Staphylococcus aureus*, a highly contagious Gram-positive bacterium, was first isolated from pus in the legs in the 1880s [1]. It exhibits considerable pathogenicity, possibly inducing conditions such as skin infections, suppuration, pneumonia, and pericarditis. The pathogenic effects of S. aureus are primarily attributed to its ability to produce invasive enzymes and toxins that infect host cells, thereby disrupting their normal growth and reproduction. As a consequence of antibiotic overuse, methicillin-resistant *Staphylococcus aureus* (MRSA) has emerged as a significant public health concern [2]. Since the first case of antibiotic-resistant strain was discovered in 1961, MRSA has shown a significant increase globally [3]. The World Health Organization (WHO) has listed MRSA as a high-priority pathogen among antimicrobial resistant bacteria, posing significant challenges to treatment due to its multidrug-resistant phenotype [4,5]. MRSA is responsible for serious clinical conditions, including bacteremia, endocarditis, skin and soft tissue infections, bone and joint infections, as well as infections associated with medical devices [6,7]. Notably, MRSA infections have caused higher hospitalization and mortality rates, compared to those caused by non-resistant S. aureus [8]. The resistance exhibited by MRSA is primarily influenced by mobile genetic elements that harbor antibiotic resistance genes. They enable the organism to resist multiple antibiotics through the production of penicillin-binding protein 2a (PBP2a) [9]. Previously, vancomycin and oral linezolid were the mainstay treatments for MRSA infections; however, strains exhibiting moderate resistance to both agents have recently emerged [10,11]. Therefore, there is an urgent need to identify and develop new therapeutic agents to inhibit MRSA effectively.

Existing research has primarily concentrated on the antibacterial effects of extracts from *Schisandra chinensis*, with the comparatively limited investigation into the antibacterial properties of its endophytic fungi. Some endophytic fungi in plants can produce metabolites, being similar to those of their host [12]. Consequently, it demonstrated a considerable practical significance in conducting antibacterial studies on extracts derived from the endophytic fungi of *Schisandra chinensis*. Plant endophytic fungi are defined as fungal communities that colonize the internal tissues and organs of plants at specific stages or throughout their life cycle, with host plants typically showing no obvious external symptoms. These endophytic fungi are prevalent in various higher plant species, encompassing both woody and herbaceous plants [13]. Gao successfully isolated a fungus from the root system of *Schisandra chinensis* in Enshi, Hubei Province, identifying it as belonging to the genus Alternaria, which displayed remarkable antibacterial properties. Under specific fermentation conditions, this fungus was able to produce active antibacterial compounds [14].

Given this context, endophytic fungi from plants are regarded as promising candidates for development into effective biocontrol agents. This study aimed to isolate a fungus from *Schisandra chinensis* and evaluate the inhibitory activity and mechanism against MRSA. We believe that this research will provide novel biocontrol fungal resources and a theoretical basis for inhibiting MRSA.

## 2. Materials and Methods

### 2.1. Isolation and Screening of Endophytic Fungi from Schisandra chinensis

Healthy roots, branches, and fruits of *Schisandra chinensis* were collected from fields in Dalian, Liaoning province, China. The samples were thoroughly washed with tap water, immersed in 75% ethanol for 3 min, and subsequently, rinsed 3 times with sterile water for 30 s, respectively [15]. Treated samples were then cut and plated onto Potato Dextrose Agar (PDA), Czapek–Dox Agar, and Fungi Agar supplemented with ampicillin. After incubating at 30 °C for 3 d, colonies that developed around the tissue were selected for purification. The isolated colonies were inoculated into their respective liquid culture media and incubated in a shaker (180 rpm/min) at 30 °C. Purified fungal strains were preserved by agar slant culture and glycerol preservation methods.

The isolated strains were inoculated into 100 mL PDB medium at a 0.1% (*v*/*v*) inoculation rate and incubated in a shaker (180 rpm/min) at 30 °C for 2 d. After incubation, the fungal suspension was centrifuged at 8000 rpm for 10 min at 4 °C, and the supernatant was collected as a sterile filtrate. This aseptic filtrate was then extracted three times with ethyl acetate, and the extracts were concentrated using a rotary evaporator (R-100, Buqi, Flawil, Switzerland). Following evaporation, 6 mL of methanol was added to dissolve the sample, which was then centrifuged at 10,000 rpm for 10 min at 4 °C to yield a crude extract of the sterile filtrate. The inhibition of MRSA by the isolated fungus was assessed by the methodology of disc diffusion method [16]. Briefly, an MRSA suspension (1 × 10^8^ CFU/mL) was mixed with LA medium at a volume ratio of 1:400 (*v*/*v*). Then, 40 mL of MRSA-containing LA was poured into a 90 mm Petri dish, where 12 mm wells were created, and 300 μL of the crude extract of the sterile filtrate was added into each well. All the experiments were performed in triplicates. Then, the Petri dishes were incubated at 37 °C for 12 h. The inhibition zones were measured, after that, a vernier caliper was used to assess the antibacterial activity of the endophytic fungi.

### 2.2. Identification of Antagonistic Fungi

The 18S rDNA sequence was amplified using the universal fungal primers ITS1 (5′-TCCGTAGGTGAACCTGCGG-3′) and ITS4R (5′-TCCTCCGCTTATTGATATGC-3′). The cycling parameters were as follows: (1) initial denaturation at 94 °C for 3 min; (2) 30 cycles of denaturation at 94 °C for 30 s, annealing at 54 °C for 30 s, and extension at 72 °C for 1.5 min; and (3) final extension at 72 °C for 10 min [17,18]. This procedure yielded a PCR product of approximate 600 bp in size. The PCR products were sent to Majorbio Technology Pharmaceutical Co., Ltd. (Shanghai, China) for sequencing. The obtained sequences were submitted to the NCBI database to determine the putative identity of the strain, and a polygene phylogenetic tree was constructed in MEGA 11.0.

### 2.3. Inhibition Spectrum of the Antagonistic Fungi

In this study, eight common pathogenic bacteria were selected for testing (Table 1). All strains were stored at the School of Life Sciences, Dalian Minzu University, China. The antagonistic fungi’s antimicrobial activity against common clinical pathogens was assessed using the assay detailed in Section 2.1.

### 2.4. Screening of Optimum Ingredient for Fermentation Medium

Rice medium, corn meal solid medium, bran solid medium, soybean meal solid medium, and PDB medium were selected as the experimental groups. Different kinds of culture medium (300 mL) were each placed into 500 mL conical flasks. A total of 15 mL of activated S10 fungal solution (1 × 10^8^ CFU/mL) was inoculated into each of the five cultures media separately and incubated in a constant temperature incubator at 32 °C for 2 d. After incubation, the cultures were homogenized with a stirring rod, followed by three rounds of ultrasonic extraction with 300 mL of ethyl acetate, each lasting 30 min. After the extraction process, the ethyl acetate layer was collected and concentrated to dryness using a rotary evaporator. The crude extract was prepared, and antibacterial experiments were conducted following the methodology described in Section 2.1, all experiments were performed in triplicate to ensure reproducibility.

### 2.5. Effects of Fermentation Parameters on the Antibacterial Activity of S10

Following the determination of the optimal culture medium composition, a single-factor test was conducted to evaluate the impact of varying fermentation conditions on the inhibition of S10. The moisture content was set at 100%, while maintaining the baseline conditions of time (2 d), pH (7.0), and temperature (32 °C). The other conditions kept constant, and only one condition was changed to conduct a single-factor test. The following conditions were set: moisture content: 50%, 100%, 150%, 200%, 250%, and 300%; time: 1.5 d, 2 d, 2.5 d, 3 d, 3.5 d, and 4 d; pH: 5.0, 6.0, 7.0, 8.0, 9.0, and 10.0; temperature: 26 °C, 28 °C, 30 °C, 32 °C, 34 °C, 36 °C, and 38 °C. Crude extract samples were subsequently produced, and the diameter of the antibacterial zone was measured by the methodology outlined in Section 2.1. Each experimental condition was repeated three times to assess the reliability of the findings.

### 2.6. Response Surface Optimization Test

Based on the single-factor test results, the four key parameters—moisture content (A), time (B), temperature (C), and pH (D)—were optimized. The response surface optimization was designed using the Box–Behnken method, implemented through Design-Expert software (v.13.0.1.0) [19], as detailed in Table 2.

### 2.7. Preparation and Separation of Antibacterial Extracts

Large-scale fermentation of strain S10 was conducted using the optimized culture medium and cultivation parameters described in Section 2.1. The resulting fermentation broth underwent sequential extraction with ethyl acetate and methanol to obtain crude extracts. The antibacterial potential of these extracts was quantitatively assessed through standardized agar diffusion assays. Subsequently, the crude extract was used for the systematic investigation of their mechanism of action.

### 2.8. Determination of Minimum Inhibitory Concentration (MIC) of S10 Against MRSA

The minimum inhibitory concentration (MIC) of the crude extract against MRSA was determined using the broth microdilution method [20]. Briefly, 100 μL of LB medium was added to each well of a 96-well plate, followed by the addition of 100 μL of crude extract (100 mg/mL) to the first well. Serial two-fold dilutions were performed to achieve a concentration gradient ranging from 50 mg/mL to 0.012 mg/mL. Subsequently, 100 μL of activated MRSA solution (1 × 10^8^ CFU/mL) was inoculated into wells containing different concentrations of the crude extract, with a total of 12 experimental groups and three replicates for each group. Controls included a positive control (MRSA inoculated in LB medium without crude extract) and a negative control (medium containing different concentrations of crude extract without MRSA). The plate was incubated in a constant temperature incubator at 37 °C for 24 h. The MIC was defined as the lowest concentration of the crude extract at which the well remained clear after incubation and upon gentle agitation.

### 2.9. Effects of the Crude Extract on the Growth Curve of MRSA

The growth curve of MRSA was determined following the method described by Shu [21] to assess the antibacterial activity of the crude extract of S10. MRSA was inoculated into LB broth at a volume ratio of 1:1000 (*v*/*v*). Subsequently, different concentrations of the crude extract were added, following the consequence named 1/2 MIC, 1 MIC, and 2 MIC. The samples were then incubated respectively in a shaker (180 rpm/min) at 37 °C for 12 h, and the MRSA was inoculated into the same amount of LB as the control group for 12 h. At hourly intervals, samples of 2 mL were collected, and absorbance at 600 nm was measured. The resulting growth curve illustrated the effect of the crude extract on MRSA, which was plotted with time as the *x*-axis and OD_600_ values as the *y*-axis.

### 2.10. Effects of Crude Extract on Alkaline Phosphatase (AKP) Content in Culture Medium

The crude extract was inoculated into the LB broth at concentrations of 1/2 MIC, 1 MIC, and 2 MIC, respectively, before inoculation with MRSA, with an extract-free medium serving as the negative control (0 MIC). Vancomycin hydrochloride (VCM) was added to the culture medium as a positive control at a final concentration of 10 μg/mL. Each concentration was tested in triplicate. Following MRSA inoculation (0.1% *v*/*v* inoculum density), cultures were incubated aerobically in a shaker (180 rpm/min) at 37 °C for 3 h. Following this, samples were centrifuged at 7000 rpm for 10 min at 4 °C to collect bacteria. AKP activity was detected using the AKP Assay Kit from Jiancheng Bioengineering Institute (Nanjing, China) and measured at a wavelength of 520 nm.

### 2.11. Effects of Crude Extract on the Membrane Potential of MRSA

To determine the MRSA membrane potential, a Rhodamine 123 fluorescence staining method was employed. The crude extract was added to MRSA during the logarithmic growth phase to achieve final concentrations of 1/2 MIC, 1MIC, and 2 MIC, with 0 MIC serving as the negative control. A 10 μg/mL concentration of VCM was introduced into the culture medium as a positive control. Three biological replicates were carried out to allow for statistical analysis of the data. The cultures were then incubated in a shaker (180 rpm) at 37 °C for 3 h, followed by centrifugation at 7000 rpm for 10 min at 4 °C to collect bacteria. The bacteria were rinsed with 0.01 mol/L phosphate-buffered saline (PBS, pH 7.2). Rhodamine 123, which was prepared as a 1 mg/mL solution in 0.01 mol/L PBS buffer, was added to the bacterial solution to achieve a final concentration of 2 μg/mL. The mixture was then incubated in the dark for 30 min, rinsed, and resuspended in 0.01 mol/L PBS. Mean fluorescence intensity (MFI) was measured using a microplate reader (H1, Agilent BioTek, Winooski, VT, USA) at an excitation wavelength of 480 nm and an emission wavelength of 530 nm, with a slit width of 10 nm. The data were expressed as MFI.

### 2.12. Determination of Biofilm Eradication Potential

The ability of the crude extract to inhibit MRSA biofilm formation was evaluated using a semi-quantitative crystal violet staining method [22]. MRSA was inoculated into a 24-well plate, and the crude extract was added to achieve final concentrations of 1/2 MIC, 1 MIC, and 2 MIC, with three replicates for each concentration. In the control group, MRSA was inoculated into an equal volume of LB in a separate 24-well plate. The plates were then incubated at 37 °C for 48 h, followed by rinsing with 0.01 mol/L PBS and fixation with 1 mL methanol. Subsequently, 500 μL of 0.1% crystal violet solution was added to all wells containing completely dried biofilms. After 30 min of dark staining, excess crystal violet was removed by washing thrice with PBS. The plates were inverted and dried in a 37 °C oven before adding 500 μL of 33% glacial acetic acid. The plates were then placed on a shaker for 30 min to dissolve the crystal violet. Finally, 50 μL of the resulting solution was transferred to a new 96-well plate for absorbance detection at 590 nm. The inhibition rate of biofilm was calculated using Equation (1) [23]:

Inhibition rate (%) = OD_positive control_ − OD_assay_/OD_positive control_ (1)

### 2.13. Determination of Effects of Crude Extract on Cell Morphology of MRSA

Scanning electron microscopy (SEM) was utilized to observe alterations in cell morphology [24]. In brief, MRSA was incubated in LB broth, and the crude extract was added to LB broth to achieve final concentrations of 1/2 MIC, 1 MIC, and 2 MIC. The sample with 0 MIC served as the control group. The cultures were then incubated in a shaker (180 rpm) at 37 °C for 4 h. The bacterial suspension was then centrifuged at 8000× *g* for 10 min at 4 °C to harvest the bacterial cells. Subsequently, the bacterial cells were washed thrice with PBS and fixed at 2.5% glutaraldehyde in a refrigerator at 4 °C overnight. After another three washes with PBS, the cells were dehydrated using ethanol concentrations of 25%, 50%, 75%, and 100% successively for 10 min each. The samples were then deposited onto silicon wafers. Following conductive coating, the morphology of the MRSA cells was examined using SEM (S-4800, Hitachi, Tokyo, Japan), the electron gun acceleration voltage was 5 kV, and the resolutions were 5 μm, 4 μm, and 1 μm, respectively, and images were collected.

## 3. Results

### 3.1. Screening and Identification of MRSA Antagonistic Fungi

**Figure 1:**
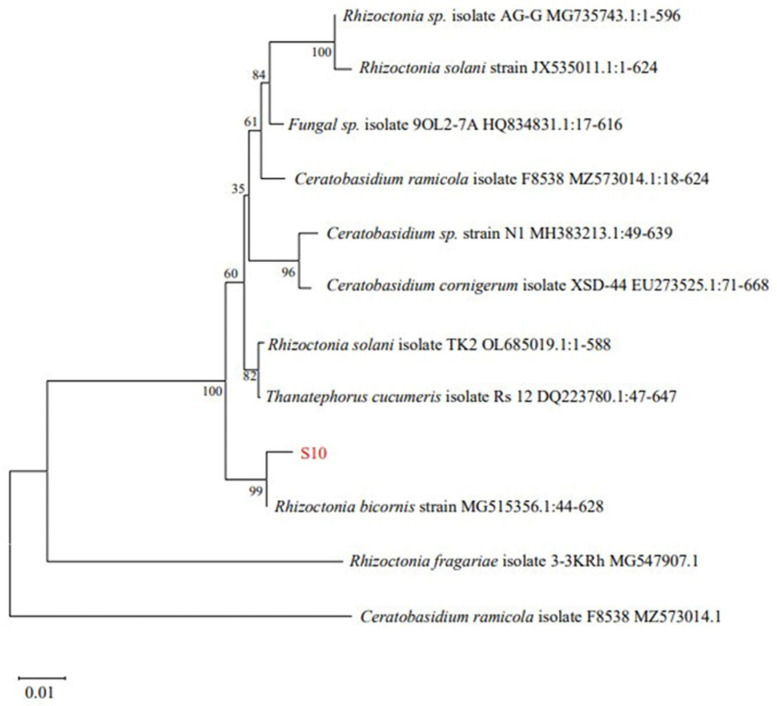
Phylogenetic tree of *Rhizoctonia bicornis* S10 based on 18S rDNA.

Twenty-one strains of endophytic fungi were isolated from *Schisandra chinensis* roots, branches, and fruit samples. Among these, only eight fungi exhibited antibacterial activity (Table 3). Among the tested samples, S10 demonstrated the most potent antibacterial activity, exhibiting an inhibition zone diameter of 18.69 ± 0.82 mm. Consequently, S10 was selected for the further investigation. Utilizing Blast on NCBI for sequence comparison revealed a significant homology between S10 and *Rhizoctonia bicornis* BNMG545356.1 at the nucleic acid level. Furthermore, employing MEGA.11 to construct the polygene phylogenetic tree, the analysis indicated that S10 and *Rhizoctonia bicornis* BN MG545356.1 clustered together on the same branch with a bootstrap value of 99% (Figure 1).

### 3.2. Inhibition Spectrum of S10

The metabolites derived from S10 exhibited inhibitory activity against eight common pathogenic bacteria (Table 4). The crude extract displayed the highest inhibitory effects on Salmonella typhimurium, with an inhibition zone measuring 18.43 ± 0.92 mm. Conversely, the crude extract showed a lower inhibitory effect on Acinetobacter baumannii, with an inhibition zone of only 13.35 ± 0.98 mm.

### 3.3. Optimization Results of Culture Medium Components

**Figure 2:**
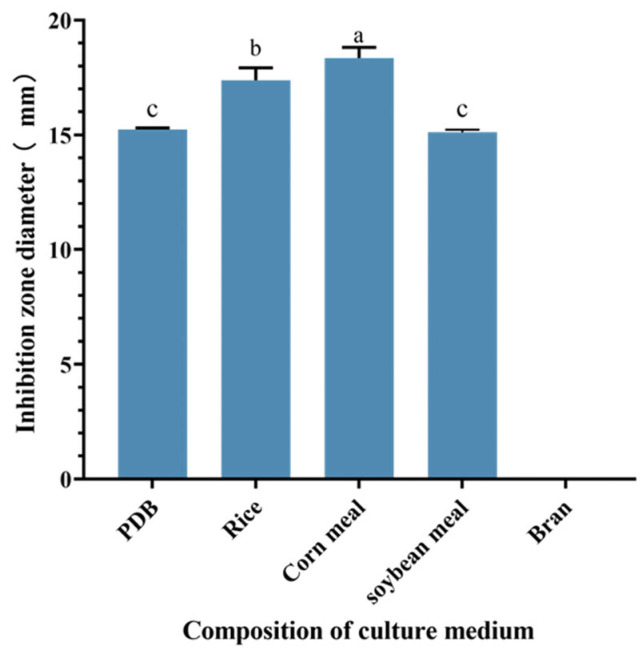
Effect of different component culture media on the inhibition zone diameter of S10 crude extract. Vertical bars represent standard error, and different letters indicate significant differences between groups (*n* = 3, *p* < 0.05).

Comparative analysis revealed variations in the anti-MRSA activity of S10 strain metabolite crude extracts across different culture conditions. The study results indicated significant differences in the inhibition zone diameters of the S10 fermentation broth among various culture media components. Specifically, when corn meal was utilized as the culture medium, the inhibition zone diameter of the crude extract measured 18.35 ± 0.46 mm, significantly surpassing those observed with other culture medium components (*p* < 0.05) (Figure 2). Consequently, corn meal was selected as a medium component for subsequent experiments.

### 3.4. Experimental Analysis of Single-Factor Effects

**Figure 3:**
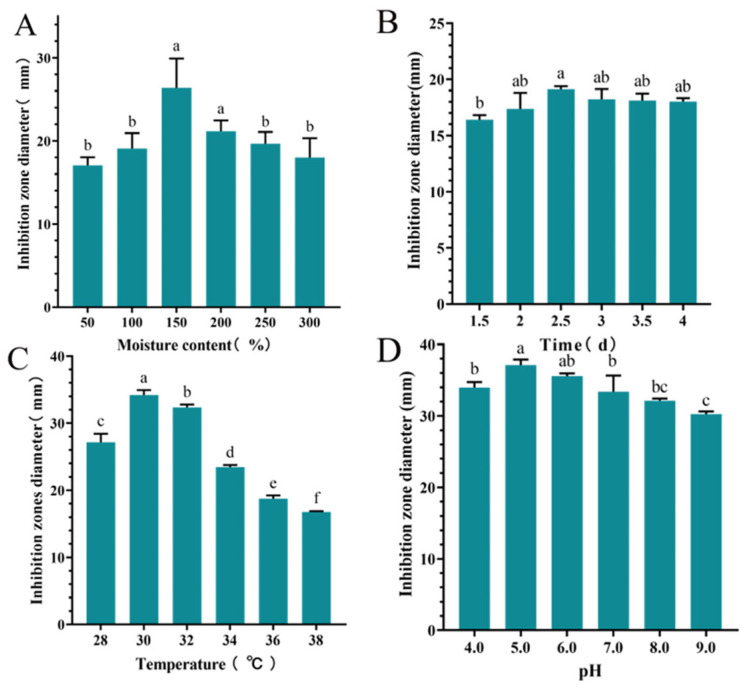
Effects of moisture content (**A**), time (**B**), temperature (**C**), and pH (**D**) on the inhibition zone diameter of the S10 crude extract. Vertical bars represent standard error, and different letters indicate significant differences between groups (*n* = 3, *p* < 0.05).

The inhibition zone diameter of S10 crude extract exhibited an initial increase followed with a decrease as the moisture content of culture medium increased. When the moisture content was 150%, the highest inhibition zone diameter recorded was 26.37 ± 3.55 mm, significantly larger than those of other groups (*p* < 0.05) (Figure 3A). Time significantly influenced the inhibition zone diameter of the S10 crude extract, with the maximum diameter of 19.11 ± 0.27 mm observed at 2.5 d (Figure 3B). The inhibition zone diameter of the S10 crude extract initially increased and then decreased with rising temperature, reaching a peak value of 34.21 ± 0.74 mm. Similarly, within the pH range of 4.0–9.0, the inhibition zone diameter of S10 crude extract demonstrated a clear pattern of initial increase followed with decrease at 30 °C (Figure 3C). Notably, at pH 5.0, the inhibition zone diameter measured 37.11 ± 0.78 mm (Figure 3D).

### 3.5. Optimization Outcomes from Response Surface Methodology Experiments

The design and results of the response surface test are presented in Table S1.

#### 3.5.1. Regression Equation and Analysis of Variance

A regression equation model was established through statistical analysis of the experimental data (Table S1) to predict the inhibition zone diameter (mm) with the following equation = 38.72 − 0.19A + 2.27B + 0.94C − 0.21D − 0.2625AB + 0.28AC + 0.67AD + 1.44BC + 0.23BD + 0.02CD − 2.55A^2^ − 5.11B^2^ − 5.68C^2^ − 3.93D^2^.

Variance analysis and a significant difference test were performed on the regression model, with the results displayed in Table S2. The regression model was statistically significant (*p* < 0.0001). A lock-of-fit value of 0.1586 (*p* > 0.05) indicated a strong correlation between actual and predicted values. The primary term B was extremely significant (*p* < 0.001), while the primary term C was significant (*p* < 0.05); however, the terms A and D were not significant. Additionally, the squared terms A2, B2, and C2 exhibited considerable antifungal activity. The coefficient of determination (R^2^ = 0.9444) signified a high correlation within the model. The F-value indicated the importance of each factor on the inhibition zone diameter, with higher F-values corresponding to greater influences. The analysis revealed that the order of influence on the inhibition zone diameter was as follows: time (B, d) > temperature (C, °C) > pH (D) > moisture content (A, %).

#### 3.5.2. Response Surface Analysis of Interaction of Various Factors

**Figure 4:**
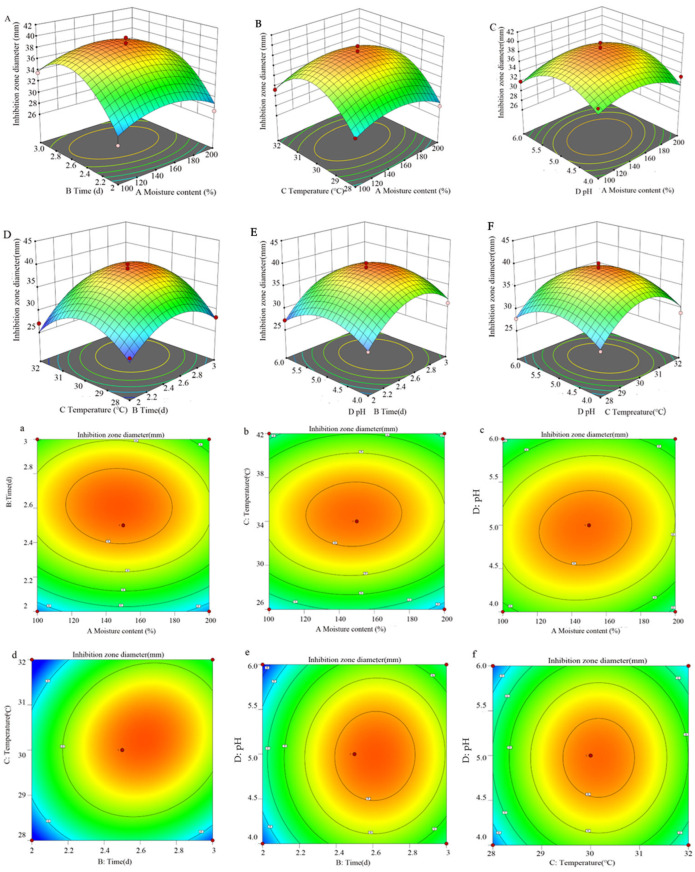
The response surface methodology and contour plots of the effects of the interaction between moisture content and time (**A**,**a**), moisture content and temperature (**B**,**b**), moisture content and pH (**C**,**c**), time and temperature (**D**,**d**), time and pH (**E**,**e**), and temperature and pH (**F**,**f**) on the inhibition zone diameter of the S10 crude extract. Note: The color gradient in the figure represents the variation in response values, with red denoting high values, yellow intermediate values, and green low values.

The response surface diagram presented in Figure 4 offers a more intuitive representation of the interaction among the four factors and their impacts on the inhibition zone diameter. The incline of the surface diagram directly correlates with the influence of the factors on the response value. A larger focal length of the contour indicates a stronger interaction between parameters. Figure 4 illustrates that the interaction between variables B (time) and C (temperature) significantly affects the antibacterial efficacy of S10. By employing a quadratic multinomial regression fitting equation, the optimal conditions were determined as follows: moisture content—147.64%; time—2.62 d; temperature—30.22 °C; pH—4.98. Under these specified conditions, the predicted inhibition zone diameter is estimated to be 39.05 mm. Furthermore, the ideal fermentation conditions involve a moisture content of 150%, a time of 2.5 d, a temperature of 30 °C, and a pH of 5.0.

### 3.6. MIC Determination of Crude Extract

As the concentration of the crude extract decreased, the optical density at 600 nm (OD600) increased, indicating an elevation in the turbidity of the culture medium in the 96-well plate. At a concentration of 6.25 mg/mL of the crude extract, the culture in the well plate appeared clear and exhibited the ability to inhibit the growth of 80% of MRSA (Table 5). Therefore, it was concluded that the MIC of the crude extract against MRSA growth was 6.25 mg/mL.

### 3.7. Effect of Crude Extract on Growth Curve of MRSA

**Figure 5:**
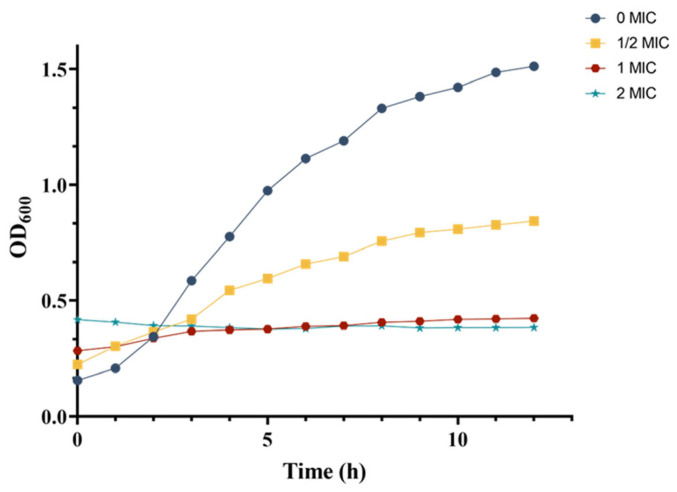
Dynamic changes in the MRSA growth curve.

The growth curves of MRSA in response to varying concentrations of crude extract were monitored by measuring optical density at 600 nm (OD_600_). In the control group, MRSA demonstrated typical growth characteristics, entering the logarithmic phase after 3 h and reaching the stationary phase by 9 h (Figure 5). In contrast, the 1/2 MIC treatment group displayed a sigmoidal growth pattern, with consistently lower OD_600_ values at each growth stage compared to those in the control group after 3 h. Notably, within the first 2 h, the OD_600_ readings in the 1/2 MIC group were higher than those of the control group, which can be attributed to the intrinsic color of the crude extract.

For the 1 MIC and 2 MIC treatment groups, the growth curves deviated from the typical S-shaped pattern, instead of appearing as nearly horizontal lines. Similar to the 1/2 MIC group, the initial OD_600_ values (before 2 h) in these groups were elevated, compared to the control and 1/2 MIC groups due to the extract’s coloration. Beyond this initial period, the OD_600_ values of the control and 1/2 MIC groups were significantly higher than those of the 1 MIC and 2 MIC groups. Furthermore, the 2 MIC group exhibited a markedly slower increase in OD_600_ than that in the 1 MIC group, with almost no observable growth.

These results demonstrated a concentration-dependent inhibitory effect of the crude extract on MRSA growth, as evidenced by the progressive reduction in OD_600_ values at each growth stage with increasing extract concentration. The growth curve analysis indicated that the crude extract significantly impaired MRSA proliferation.

### 3.8. Results of AKP Content Determination

**Figure 6:**
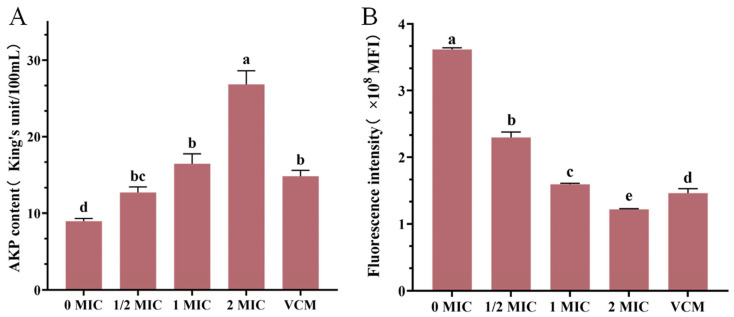
AKP content (**A**), the effect of crude extract on the membrane potential of MRSA (**B**). Vertical bars represent standard error, and different letters indicate significant differences between groups (*n* = 3, *p* < 0.05).

The experimental data revealed a concentration-dependent relationship between crude extract and MRSA membrane integrity (Figure 6), at the 1 MIC concentration of the crude extract, AKP levels in the culture medium exceeded those observed in the positive control group. AKP levels in MRSA fermentation broth showed progressive elevation with increasing extract concentrations (Figure 6A), suggesting enhanced bacterial membrane permeability and the subsequent leakage of intracellular components.

### 3.9. Results of Membrane Potential Measurement

The administration of the crude extract induced marked fluctuations in the membrane potential of MRSA strains (Figure 6B). The negative control group maintained an average fluorescence intensity of 3,627,594 MFI, while treatment groups exhibited marked reductions, as follows: 2,297,638 MFI (1/2 MIC), 1,592,686 MFI (1 MIC), and 1,217,434 MFI (2 MIC). Treatment with crude extract at 1 MIC produced comparable fluorescence intensity to the positive control (1, 459, 356), with no significant difference. These values corresponded to concentration-dependent decreases of 36.66%, 56.10%, and 66.44% relative to controls, respectively. Further analysis revealed a 30.68% reduction in fluorescence intensity between 1/2 MIC and 1 MIC groups, with an additional 23.56% decrease observed at 2 MIC, compared to 1 MIC concentrations. These findings collectively demonstrated that crude extract treatment induced membrane potential collapse in MRSA, the disruption of cellular homeostasis, and ultimate bactericidal effects through compromised metabolic processes.

### 3.10. Effects of Crude Extract on MRSA Biofilm Formation

The inhibition rate of MRSA biofilm formation gradually increased with the concentration of crude extract (Table 6). At a concentration equivalent to 1/2 MIC, the crude extract was able to inhibit biofilm formation. Moreover, at the 2 MIC concentration, the inhibition rate peaked, resulting in the inhibition of nearly 50% of biofilm formation. Therefore, the crude extract of S10 has the potential to inhibit MRSA biofilm formation.

### 3.11. Effects of Crude Extract on Cell Morphology of MRSA

**Figure 7:**
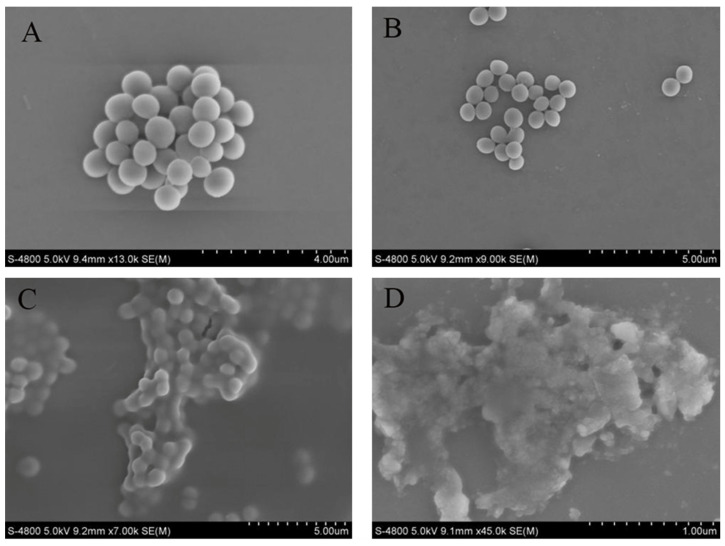
Sample to morphology of MRSA. Control group (**A**); 1/2 MIC (**B**); 1 MIC (**C**); 2 MIC (**D**).

SEM observations confirmed the detrimental effects of the crude extract on MRSA cells (Figure 7). In the control group, the membranes of MRSA cells were complete and intact, exhibiting bacteria in a spherical shape and a dense packing arrangement. When 1/2 MIC of the crude extract was added, the morphology of MRSA cells remained largely unchanged, retaining their spherical shape; however, compared to the control group, cell density decreased, and the arrangement became more dispersed. With an increasing concentration of crude extract, profound alterations in the appearance of MRSA cells were observed: the bacteria exhibited signs of breakage, deformation, shrinkage, and aggregation. These changes were notably pronounced at a concentration of 2 MIC.

## 4. Discussion

At present, the widespread use of antibiotics has resulted in the emergence of drug-resistant bacteria. There is an urgent need to discover new antibiotics as alternatives to conventional antibiotic treatments. In contrast to chemically synthesized drugs, *Schisandra chinensis* emerges as a promising natural bioactive agent, being distinguished by its superior safety profile, low toxicity, as well as effects on reducing likelihood of inducing drug resistance. These attributes position it as a valuable candidate for developing alternatives to conventional antibacterial drugs [25,26]. Among its bioactive constituents, lignin compounds have garnered significant attention as key pharmacological components, with their properties and applications being documented in recent studies [27,28,29]. Current research on their antibacterial activity primarily focuses on their efficacy against microbial communities, such as foodborne pathogens and skin microbiota [30]. Notably, Jae-Hee Jeong [31] successfully isolated and characterized antibacterial compounds from *Schisandra chinensis*, demonstrating their inhibitory effects against *Streptococcus mutans* KCCM 40105, with tartaric acid identified as the primary active antibacterial component. Despite these advancements, research on endophytic fungi associated with *Schisandra chinensis* remains relatively underexplored. Previous investigations have revealed that certain endophytic fungi derived from *Schisandra chinensis* exhibit notable antibacterial properties [16]. In this study, we isolated multiple endophytic fungal strains from *Schisandra chinensis*, several of which demonstrated significant antibacterial activity against MRSA. Among these, S10 exhibited particularly potent antibacterial effects and was preliminarily identified through 18S rDNA sequencing. These findings provide crucial theoretical foundations for the development of novel antibiotics targeting MRSA, highlighting the untapped potential of endophytic fungi in *Schisandra chinensis* as a source of bioactive compounds.

The selection of an appropriate culture medium and precise fermentation conditions are paramount for the growth and activity of microorganisms. In this investigation, it was found that the crude extract of S10 exhibited the most significant inhibitory effect on MRSA when corn meal was utilized as the culture medium. Various fermentation parameters were assessed for their impact on the inhibitory potential of the S10 crude extract, with time demonstrating the strongest influence, followed by temperature, pH, and moisture content. The optimal fermentation settings were determined to be a moisture content of 150%, a time of 2.5 d, a temperature of 30 °C, and a pH of 5.0. Notably, under these optimized conditions, the inhibition zone against MRSA measured 36.88 mm in diameter.

The cell membrane plays a crucial role as a protective structure, serving as a permeability barrier for the passage of small ions, while also ensuring the stability of intracellular components like nucleic acid, enzymes, and proteins within the cell [32]. AKP is predominantly present intracellularly and is scarce in fermentation broth [33]. A reduction in the integrity of the cell membrane triggers an irreversible apoptosis process, thereby initiating the cell death [34]. Past studies have demonstrated that bacteria cells undergo alterations in membrane permeability under the influence of antibacterial agents, resulting in the release of intracellular substances [35,36]. In our investigation, the addition of crude extract at varying concentrations led to an increase in AKP levels in MRSA fermentation broth and a subsequent decrease in membrane potential, aligning with findings from existing literature. The SEM analysis further confirmed that the crude extract disrupted the cell membrane of MRSA. Consequently, it is hypothesized that crude extract of S10 inhibited MRSA growth by compromising its cell membrane integrity.

Biofilms are primarily composed of self-produced extracellular polymeric substances (EPS), which act as a structural scaffold to encase bacteria on surfaces and shield them from environmental stressors. These microbial biofilms firmly attach to both biological and non-biological surfaces, providing a protective barrier that enhances the survival of microorganisms under extreme conditions. This protection extends to environmental challenges, the effects of antibiotics and antifungal agents, and the defensive responses of the host immune system during infections [37,38]. Microorganisms within biofilms exhibit greater resistance compared to their planktonic counterparts [39]. In this study, crystal violet assays revealed that with increasing concentrations of the crude extract, there was a more pronounced inhibitory effect on biofilm formation. Notably, at a concentration of 2 MIC of the crude extract, a 50% reduction in biofilm formation was observed. These results suggested that the crude extract S10 may attenuate the pathogenicity and drug resistance of MRSA by disrupting MRSA biofilm formation.

To the best of our knowledge, the majority of research on the antibacterial properties of *Schisandra chinensis* has predominantly focused on its fruits, with a relatively limited investigation into the antibacterial properties of *Schisandra chinensis* endophytic fungi. In this study, we isolated a fungus demonstrating significant antibacterial efficacy against MRSA and enhanced its effectiveness through modifications to the culture medium and fermentation conditions. Furthermore, we conducted an assessment of the antibacterial mechanism exhibited by the S10 crude extract on MRSA, thereby offering a foundational basis for future investigations into novel antibiotics for combating MRSA.

## 5. Conclusions

In this study, we isolated an endophytic fungus from *Schisandra chinensis* that exhibits potent antibacterial activity against MRSA. This strain exhibited broad-spectrum antimicrobial properties and was identified as *Rhizoctonia bicornis* S10. The crude extract of S10 exhibited the most significant inhibitory effect on MRSA when corn meal was utilized as the culture medium. The fermentation factors affecting the inhibitory effect of S10 fermentation broth were time > temperature > pH > moisture content, and the optimal fermentation conditions were a moisture content of 150%, a time of 2.5 d, a temperature of 30 °C, and pH of 5.0. The inhibition zone diameter was 36.88 mm under these conditions. The crude extract of S10 could effectively inhibit the growth of MRSA by disrupting its cell membrane, leading to increased AKP content in the supernatant and a reduction in membrane potential. Additionally, it suppressed the formation of MRSA biofilms. In conclusion, I believe that this study will provide a foundation for the future development of novel anti-MRSA therapeutic agents.

## Figures and Tables

**Table 1:** Pathogens used for inhibition spectrum research.

Pathogen	Type of Culture Medium
*Staphylococcus aureus*	LB Broth
*Escherichia coli*	LB Broth
*Vibrio parahaemolyticus*	LB Broth
ESBL *E. coli*	LB Broth
*Pseudomonas aeruginosa*	LB Broth
*Salmonella typhimurium*	LB Broth
*Klebsiella pneumoniae*	Nutrient Broth
*Acinetobacter baumannii*	Brian Heart Infusion

**Table 2:** Factors and levels of Box–Behnken tests.

Levels	Factors			
	A Moisture Content (%)	B Time (d)	C Temperature (°C)	D pH
−1	100	2	28	4.0
0	150	2.5	30	5.0
1	200	3	32	6.0

**Table 3:** Screening results of endophytic fungi against MRSA.

Strain Number	Inhibition Zone Diameter (mm)	Strain Number	Inhibition Zone Diameter (mm)
S10	18.69 ± 0.82	S1	17.02 ± 0.79
W1	15.57 ± 1.67	S2	14.73 ± 0.81
W2	17.33 ± 0.46	W8	16.73 ± 0.24
W4	17.89 ± 1.12	W7	13.62 ± 0.18

**Table 4:** Inhibition spectrum of S10.

Pathogen	Inhibition Zone Diameter (mm)	Pathogen	Inhibition Zone Diameter (mm)
*Staphylococcus aureus*	14.15 ± 0.29	ESBL *E. coli*	17.55 ± 1.22
*Acinetobacter baumannii*	13.35 ± 0.98	*Escherichia coli*	13.93 ± 0.42
*Vibrio parahaemolyticus*	16.64 ± 0.78	*Klebsiella pneumoniae*	17.37 ± 0.32
*Pseudomonas aeruginosa*	16.85 ± 0.19	*Salmonella typhimurium*	18.43 ± 0.92

**Table 5:** Inhibition of MRSA by crude extracts of different concentrations.

Concentration of Crude Extract (μg/mL)	OD_600_	Concentration of Crude Extract (μg/mL)	OD_600_
50.00	0.030 ± 0.011	1.56	0.371 ± 0.031
25.00	0.080 ± 0.007	0.78	0.482 ± 0.078
12.5	0.122 ± 0.019	Negative control	0.047 ± 0.003
6.25	0.165 ± 0.009	Positive control	0.827 ± 0.011

**Table 6:** Sample inhibited biofilm of MRSA.

Concentration of Crude Extract (μg/mL)	OD_590_	Inhibition Rate (%)
0 (Control)	1.233 ± 0.041	-
1/2 MIC	1.043 ± 0.038	15.4%
1 MIC	0.694 ± 0.052	42.7%
2 MIC	0.635 ± 0.047	48.4%

## Data Availability

The data analyzed in this study are included within the paper.

## Supplementary Materials

The following supporting information can be downloaded at: https://www.mdpi.com/article/10.3390/microorganisms13050982/s1, Table S1: Experimental design and results of strain culture conditions. Table S2: Regression analysis of experimental results based on the Box-Behnken design.

## References

[1] Newsom S.W.B. (2008). Ogston’s coccus. J. Hosp. Infect..

[2] Cameron J.K., Hall L., Tong S.Y.C., Paterson D.L., Halton K. (2019). Incidence of community-onset MRSA in Australia: Least reported where it is most prevalent. Antimicrob. Resist. Infect. Control.

[3] Cheng C., Wu Z., McClements D.J., Zou L., Liu W. (2019). Improvement on stability, loading capacity and sustained release of rhamnolipids modified curcumin liposomes. Colloids Surf. B Biointerfaces.

[4] Tacconelli E., Carrara E., Savoldi A., Harbarth S., Mendelson M., Monnet D.L., Pulcini C., Kahlmeter G., Kluytmans J., Carmeli Y. (2018). Discovery, research, and development of new antibiotics: The WHO priority list of antibiotic-resistant bacteria and tuberculosis. Lancet Infect. Dis..

[5] Roy A., Poddar N., Panigrahi K., Pathi B., Nayak S.R., Dandapat R., Pattnaik D., Praharaj A.K., Patro A.R.K. (2023). Evaluation of In-Vitro Activity of Ceftaroline Against Methicillin-Resistant *Staphylococcus aureus* Clinical Isolates. Cureus.

[6] Alan H., Cian O. (2018). Emerging nanomedicine therapies to counter the rise of methicillin-resistant *Staphylococcus aureus*. Materials.

[7] Ortwine J.K., Bhavan K. (2020). Morbidity, mortality, and management of methicillin-resistant *S. aureus* bacteremia in the US: Update on antibacterial choices and understanding. Hosp. Pract..

[8] Turnidge J.D., Kotsanas D., Munckhof W., Roberts S., Bennett C.M., Nimmo G.R., Coombs G.W., Murray R.J., Howden B., Johnson P.D.R. (2009). *Staphylococcus aureus* bacteremia: A major cause of mortality in Australia and New Zealand. Med. J. Aust..

[9] Peacock S.J., Paterson G.K. (2015). Mechanisms of methicillin resistance in *Staphylococcus aureus*. Annu. Rev. Biochem..

[10] Yoneda A., Thänert R., Burnham C.A.D., Dantas G. (2020). In vitro activity of meropenem/piperacillin/tazobactam triple combination therapy against clinical isolates of *Staphylococcus aureus*, *Staphylococcus epidermidis*, *Staphylococcus pseudintermedius*, and vancomycin-resistant *Enterococcus* spp.. Int. J. Antimicrob. Agents.

[11] Chen X.H., Liu P.P., Luo X.F., Huang A.L., Wan J.Q. (2024). Study on the antibacterial activity and mechanism of cinnamaldehyde against methicillin-resistant *Staphylococcus aureus*. Eur. Food Res. Technol..

[12] Qin D., Wang L., Han M., Wang J., Song H., Yan X., Duan X., Dong J. (2018). Effects of an Endophytic Fungus *Umbelopsis dimorpha* on the Secondary Metabolites of Host–Plant *Kadsura angustifolia*. Front. Microbiol..

[13] Hassan A.A., Abdessamad D., Peter P. (2011). Fungal endophytes: Unique plant inhabitants with great promises. Appl. Microbiol. Biotechnol..

[14] Ying G., Jia Z., Hanli R. (2020). Trichothecenes from an Endophytic Fungus *Alternaria* sp. sb23. Planta Medica.

[15] Xu W., Zhang L.-Y., Goodwin P.-H., Xia M.-C., Zhang J., Wang Q., Liang J., Sun R.-H., Wu C., Yang L.-R. (2020). Isolation, identification, and complete genome assembly of an endophytic *Bacillus velezensis* YB–130, potential biocontrol agent against *Fusarium graminearum*. Front. Microbiol..

[16] Abdelgawwad M.R., Mahmood A., Farraj DA A., El-Abedein AI Z., Mahmoud A.H., Bukhari S.M. (2020). In-vitro Antimicrobial Activities of *Solanum villosum* (L.) Lam; Crude Extract Solvent Comparison. J. King Saud Univ.-Sci..

[17] Santos S.N., Ferraris F.K., Souza A.O., Henriques MD G., Melo I.S. (2012). Endophytic fungi from *combretum leprosum* with potential anticancer and antifungal activity. Symbiosis.

[18] Guenter S., Gorkiewicz G., Halwachs B., Kashofer K., Thueringer A., Wurm P., Zollner-Schwetz I., Valenti T., Prattes J., Wunsch S. (2020). Impact of ITS-based sequencing on antifungal treatment of patients with suspected invasive fungal infections. J. Fungi.

[19] He J., Zhang X., Wang Q., Li N., Ding D., Wang B. (2023). Optimization of the fermentation conditions of *Metarhizium robertsii* and its biological control of wolfberry root rot disease. Microorganisms.

[20] Schn T., Werngren J., Machado D., Borroni E., Cambau E. (2021). Multicentre testing of the EUCAST broth microdilution reference method for MIC determination on *Mycobacterium tuberculosis*. Clin. Microbiol. Infect..

[21] Shu Q., Niu Y.-W., Zhao W.-J., Chen Q.-H. (2019). Antibacterial activity and mannosyl erythritol lipids against vegetative cells and spores of *Bacillus cereus*. Food Control.

[22] Xu Z.B., Liang Y.R., Lin S.Q., Chen D.Q., Li B., Li L., Deng Y. (2016). Crystal violet and XTT assays on *Staphylococcus aureus* biofilm quantification. Curr. Microbiol..

[23] Bakkiyaraj D., Nandhini J.R., Malathy B., Pandian S.K. (2013). The anti-biofilm potential of pomegranate (*Punica granatum* L.) extract against human bacterial and fungal pathogens. Biofouling.

[24] Cui H.-Y., Bai M., Lin L. (2018). Plasma–treated poly (ethylene oxide) nanofibers containing tea tree oil/beta-cyclodextrin inclusion complex for antibacterial packaging. Carbohydr. Polym..

[25] Zheng H.F., Liu Y., Cai J., Zhang M., Wen Y., Guo L. (2022). The exploration of anti-*Vibrio parahaemolyticus* substances from *Phellodendri Chinensis Cortex* as a preservative for shrimp storage. Front. Microbiol..

[26] Lei G., Jiacai G., Fuquan X. (2017). Optimized extraction process and identification of antibacterial substances from Rhubarb against aquatic pathogenic *Vibrio harveyi*. 3 Biotech.

[27] Kim T.I., Choi E.J., Chung C.P., Han S.B., Ku Y. (2002). Antimicrobial effect of Zea mays L. and Magnoliae cortex extract mixtures on periodontal pathogen and effect on human gingival fibroblast cellular activity. J. Korean Acad. Periodontol..

[28] Jeong P.H., Kim Y.S., Shin D.H. (2006). Changes of physicochemical characteristics of during postharvest ripening at various temperatures. Korean J. Food Sci. Technol..

[29] Nam H.H., Kim H.J., Choi N.J., Roh S.S., Choo B.K. (2015). A comparison of antioxidant activity from *Schisandra chinensis* water extracts depending on stir-frying and stir-frying with liquids process. Korean J. Org. Agric..

[30] Kim J.H., Kim M.J., Choi S.K., Bae S.H., An S.K., Yoon Y.M. (2011). Antioxidant and antimicrobial effects of lemon and eucalyptus essential oils against skin floras. J. Soc. Cosmet. Sci. Korean.

[31] Jeong J.H., Kim S.H., Huh C.K. (2023). Separation and identification of an antimicrobial substance from *Schisandra chinensis* extract against *Streptococcus mutans* KCCM 40105 Strain. Molecules.

[32] Chen C.Z., Cooper S.L. (2002). Interactions between dendrimer biocides and bacterial membranes. Biomaterials.

[33] Xing K., Chen X.-G., Kong M., Liu C.-S., Cha D.-S., Park H.-J. (2009). Effect of oleoyl–chitosan nanoparticles as a novel antibacterial dispersion system on viability, membrane permeability and cell morphology of *Escherichia coli* and *Staphylococcus aureus*. Carbohyd. Polym..

[34] Bensassi F., Gallerne C., Dein OS E., Hajlaoui M.R., Bacha H., Lemaire C. (2011). Mechanism of alternariol monomethyl ether-induced mitochondrial apoptosis in human colon carcinoma cells. Toxicology.

[35] Xue Y.-B., Yang M.-G., Li S.-H., Li Z.-J., Liu H.-H., Guo Q.-B., Wang C.-L. (2019). The antibiotic activity and mechanisms of active metabolites (*Streptomyces alboflavus* TD–1) against *Ralstonia solanacearum*. Biotechnol. Lett..

[36] Wang B.C., Guo Y.S., Chen X.T., Ma J.L., Lei X., Wang W.Z., Long Y.H. (2023). Assessment of the Biocontrol Potential of *Bacillus velezensis* WL–23 against Kiwifruit Canker Caused by *Pseudomonas syringae* pv. *actinidiae*. Int. J. Mol. Sci..

[37] Thi M.T.T., Wibowo D., Rehm B.H.A. (2020). *Pseudomonas aeruginosa* Biofilms. Int. J. Mol. Sci..

[38] Atiencia-Carrera M.B., Cabezas-Mera F.S., Tejera E., Machado A. (2022). Prevalence of biofilms in *Candida* spp. bloodstream infections: A meta-analysis. PLoS ONE.

[39] Marsh P.D., Head D.A., Devine D.A. (2015). Dental plaque as a biofilm and a microbial community—Implications for treatment. BMC Oral Health.

