# Supplementary material for: Optimization of Fermentation Conditions for Endophytic Fungi from Schisandra chinensis and Investigation of Their Antibacterial Mechanisms Against Methicillin-Resistant Staphylococcus aureus
Source: Microorganisms. 2025 Apr 25;13(5):982. doi: 10.3390/microorganisms13050982 (PMC12114053; doi:10.3390/microorganisms13050982)
Supplement: Supplementary file 1 [file microorganisms-13-00982-s001.zip › microorganisms-3557266-supplementary.pdf]

Optimization of Fermentation Conditions for Endophytic Fungi from *Schisandra chinensis* and Investigation of Their Antibacterial Mechanisms Against Methicillin-Resistant *Staphylococcus aureus*

Mengyu Li [1], Yuewei Shi [1], Wenwei Ma [1], Shouyuan Cai [1], Xinyuan Yang [1], Lukai Xu [1], Xiyan Hou [1], Lulu Wang [1], Liming Jin [1] * and Chunshan Quan [1] *

[1] Key Laboratory of Biotechnology and Bioresources Utilization of Ministry of Education, College of Life Sciences, Dalian Minzu University Dalian 116600, China; 15825315619@163.com (M.L.); 1215969297@qq.com (Y.S.); 2164877320@qq.com (W.M.); 2804844280@qq.com (S.C.); 1439033253@qq.com (X.Y.); 1565604834@qq.com (L.X.); xyhous@dlnu.edu.cn; (X.H.); wanglulu0813@126.com (L.W.)

* Correspondence: jlm@dlnu.edu.cn (L.J.); mikyeken@dlnu.edu.cn (C.Q.); Tel.: +86-13504088982 (L.J.); +86-18609864172 (C.Q.)

Table S1 Experimental design and results of strain culture conditions

NO.	A Moisture content (%)	B Time (d)	C Temperature (℃)	D pH	Inhibition Zone Diameter (mm)
1	-1	-1	0	0	27.19
2	1	-1	0	0	27.51
3	-1	1	0	0	33.57
4	1	1	0	0	32.84
5	0	0	-1	-1	27.95
6	0	0	1	-1	28.83
7	0	0	-1	1	27.77
8	0	0	1	1	28.71
9	-1	0	0	-1	34.47
10	1	0	0	-1	33.39
11	-1	0	0	1	32.03
12	1	0	0	1	33.61
13	0	-1	-1	0	27.03
14	0	1	-1	0	28.18
15	0	-1	1	0	27.08
16	0	1	1	0	33.97
17	-1	0	-1	0	30.11
18	1	0	-1	0	28.35

19	-1	0	1	0	31.37
20	1	0	1	0	30.73
21	0	-1	0	-1	27.66
22	0	1	0	-1	30.98
23	0	-1	0	1	27.24
24	0	1	0	1	31.46
25	0	0	0	0	40.05
26	0	0	0	0	38.03
27	0	0	0	0	38.49
28	0	0	0	0	37.9
29	0	0	0	0	39.13

Table S2 Regression analysis of experimental results based on the Box- Behnken design

Source	Sum of squares	df	Mean Square	F-value	p-value	Significance
Model	439.17	14	31.37	16.98	<0.0001	**
A	0.4447	1	0.4447	0.2408	0.6313	
B	62.06	1	62.06	33.60	<0.0001	**
C	10.64	1	10.64	5.76	0.0309	
D	0.5043	1	0.5043	0.2731	0.6095	
AB	0.2756	1	0.2756	0.1492	0.7051	
AC	0.3136	1	0.3136	0.1698	0.6865	
AD	1.77	1	1.77	0.9578	0.3444	
BC	8.24	1	8.24	4.46	0.0532	
BD	0.2025	1	0.2025	0.1096	0.7455	
CD	0.0009	1	0.0009	0.0005	0.9827	
A^2	42.12	1	42.12	22.81	0.0003	
B^2	169.10	1	169.10	91.56	<0.0001	**
C^2	209.61	1	209.61	113.49	<0.0001	**
D^2	100.29	1	100.29	54.30	<0.0001	**
Residual	25.86	14	1.85			
Lock of Fit	22.72	10	2.27	2.90	0.1586	
Pure of Error	3.14	4	0.7846			
Cor Total	465.03	28				
		R^2=0.9444	R^2_{adj}=0.8888			
